# A Theory-Based Approach to Predict Stress Relaxation Behavior Among South Asian Americans: A Cross-Sectional Study

**DOI:** 10.3390/ijerph23020253

**Published:** 2026-02-17

**Authors:** Manoj Sharma, Asma Awan, Vikash Patel, Badrunnisa Hanif, Aastha Poudel, Tooba Laeeq, Sandhya Wahi-Gururaj

**Affiliations:** 1 Department of Social and Behavioral Health, School of Public Health, University of Nevada, Las Vegas, Las Vegas, NV 89154, USA; manoj.sharma@unlv.edu (M.S.); patelv8@unlv.nevada.edu (V.P.); poudea1@unlv.nevada.edu (A.P.); 2 Department of Internal Medicine, Kirk Kerkorian School of Medicine, University of Nevada, Las Vegas, Las Vegas, NV 89102, USA; badrunnisa.hanif@unlv.edu (B.H.); tooba.laeeq@unlv.edu (T.L.); sandhya.wahi@unlv.edu (S.W.-G.)

**Keywords:** mental health, work–life balance, immigration, depression, anxiety, intergenerational conflict, acculturation

## Abstract

**Highlights:**

**Public health relevance—How does this work relate to a public health issue?**

- Stress relaxation techniques are crucial for South Asian Americans due to high rates of untreated anxiety, depression, and acculturative stress from immigration and racism, which exacerbate mental health disorders.
- This work relates to public health by preventing stress-linked physical conditions like hypertension and diabetes, reducing overall disease burden and healthcare utilization in this population.

**Public health significance—Why is this work of significance to public health?**

- Addressing stress relaxation and mental health is significant for South Asian Americans, as cultural stigma and underutilization of services lead to widespread unaddressed emotional distress and mood disorders affecting 1 in 5 individuals.
- This work holds public health significance by promoting health equity, mitigating the links between chronic stress and physical illnesses, and fostering community well-being in a growing demographic prone to misinformation and religious tensions impacting mental health.

**Public health implications—What are the key implications or messages for practitioners, policy makers and/or researchers in public health?**

- Key implications include the need for practitioners to develop culturally tailored stress relaxation interventions to overcome barriers like stigma and improve access to mental health services for South Asian Americans.
- For policymakers and researchers, implications involve funding targeted programs and conducting more studies on severe mental conditions to address data gaps, underreporting, and socioeconomic factors driving disparities in this population.

**Abstract:**

South Asian Americans experience multifaceted sociocultural and acculturative stressors that influence mental well-being, yet few studies have applied contemporary behavioral theories to understand relaxation behaviors in this population. This cross-sectional study examined predictors of initiating and sustaining relaxation behaviors using the Multi-Theory Model (MTM) of health behavior change. A web-based survey of 271 South Asian adults incorporated the Perceived Stress Scale (PSS-10), MTM constructs, and sociodemographic characteristics. Reliability was high across MTM subscales (Cronbach’s α = 0.81–0.93). Structural equation modeling demonstrated acceptable fit (CFI > 0.90, TLI > 0.90, RMSEA < 0.08, SRMR < 0.08). Hierarchical regressions revealed that among participants practicing relaxation (*n* = 202), behavioral confidence significantly predicted initiation (β = 0.481, *p* < 0.001), followed by participatory dialogue (β = 0.194, *p* < 0.05) and changes in the physical environment (β = 0.242, *p* < 0.01). Emotional transformation strongly predicted sustenance (β = 0.395, *p* < 0.001), along with practice for change (β = 0.307, *p* < 0.05) and changes in the social environment (β = 0.210, *p* < 0.05). MTM constructs explained 69.8% of initiation variance and 70.4% of sustenance variance. Among non-practitioners, participatory dialogue predicted initiation (β ≈ 0.18–0.34, *p* < 0.05), and emotional transformation predicted sustenance (β = 0.570, *p* < 0.001). These findings underscore MTM’s strong predictive utility and support culturally tailored interventions enhancing confidence, emotional regulation, and social/environmental supports to promote relaxation behaviors in South Asian communities in the United States.

## 1. Introduction

The World Health Organization (WHO) defines stress as a state of worry or mental strain caused by challenging circumstances [1]. Stress is a significant public health concern that affects individuals across various professions, age groups, and backgrounds. It is negatively associated with income, being female, and being single [2]. Smith and Wesselbaum [2] also noted that stress follows an inverted U-shaped pattern with age, peaking at 39 years. A recent worldwide survey by the Worldwide Independent Network (WIN) indicated that approximately 79% of individuals experience stress to varying degrees in their daily lives, driven largely by factors such as work and financial concerns [3].

As of 2010, South Asian Americans (SAAs) represented approximately 20% of the overall Asian American population in the United States [4,5]. These populations experience stress related to acculturation, feelings of alienation, language barriers, immigration status, family expectations, intergenerational conflicts, and discrimination [6]. In particular, first-generation SAAs face communication challenges that lead to social isolation and psychological distress, whereas the children of SAAs experience stress due to biculturalism. Studies also indicate that South Asian women are especially vulnerable to stress due to exposure to violence and the effects of patriarchal community structures [6]. Of the listed stressors, intergenerational conflict remains one of the most significant sources of stress across South Asian families [7].

Similarly, the stress faced by SAAs leads to consequences such as self-harm and mental illnesses, including depression, anxiety, insomnia, and cardiovascular disease. It is also linked to the manifestation of stress as physical symptoms (somatization) and risk of cardiovascular diseases [2,6]. Previous population-based research has reported associations between perceived stress levels and sociodemographic characteristics such as income, gender, and marital status [5,6,7]. These findings suggest that structural and social contexts, including financial strain, caregiving roles, and social support availability may influence stress experiences. However, these associations are not deterministic and may vary across time and cultural settings.

### 1.1. Importance of Work–Life Balance

Work–life balance (WLB) is increasingly seen as an essential aspect of health and well-being. It refers to a degree of harmony between professional obligations and personal life commitments, affecting mental health, job satisfaction, and overall productivity [8]. Many South Asians residing in the United States are engaged in demanding careers and maintain close ties to traditional family structures, making WLB not only essential but deeply complex [9]. Cultural norms, gender roles, and immigration-related challenges may collectively shape how this population experiences, negotiates, and copes with stressors related to both work and family life.

Gender roles and family expectations have a particular influence on South Asians’ experience with WLB. Traditional gender norms persist as more females take on responsibilities outside the home. Although extended family members can provide essential childcare assistance [10], these arrangements may also impose expectations that limit women’s autonomy [11]. While both South Asian and American women encounter work–life balance issues, Adya [12] indicates that South Asian women suffer more long-term adverse career effects, likely attributable to cultural limitations and lower institutional support. South Asian women in academia describe a bicultural negotiation process to manage their identities while excelling in their careers [13].

Cultural adaptations, religious involvement, and immigration cannot be overlooked as significant forces for sustaining WLB. Immigrant professionals often employ strategies such as code-switching and selective assimilation to navigate US workplace norms while maintaining cultural heritage [14]. Religious involvement further provides a vital coping resource, helping individuals reconcile work and family demands with personal values [15]. Furthermore, visa and immigration policies significantly affect WLB, particularly for those on H-1B and H-4 visas [16].

### 1.2. Determinants of Stress and Relaxation Behavior

Stress among South Asians living in the US is shaped by a complex interplay of sociocultural, economic, environmental, and psychological determinants. At the core, family expectations and obligations, as well as academic or professional excellence, serve as persistent stressors, especially among women and young professionals, respectively [17,18]. Low socioeconomic conditions for South Asians, particularly men, contribute significantly to chronic stress, primarily through factors such as financial instability, unemployment, and access to basic resources [19].

Cultural dissonance, value clashes between parents and children, identity ambiguity, and the perceived pressure to “fit in” collectively contribute to significant psychological stress among South Asians traversing bicultural environments [20]. The notion of having cognitive dissonance between home values aligned with workplace expectations compounds daily stress [21]. Middle-aged men, especially immigrants, have heightened psychological distress linked to acculturative challenges, stigma around mental health, and higher rates of untreated substance use disorders [22]. Patriarchal customs, unequal power structures, and expectations to uphold family honor adversely impact South Asian women, contributing to stressors such as domestic abuse, forced isolation, financial coercion, and fear of social stigma [20]. Midlife women in Canada frequently encounter societal and familial expectations of being dutiful wives and mothers while also engaging in professional work [23].

### 1.3. Practices of Relaxation Behavior

South Asians use various culturally rooted strategies to manage stress, including religious practices, social support, and cultural affiliation. Religious involvement provides a vital coping resource, helping individuals reconcile work and family demands with personal values [15]. Prayer, yoga, and gratitude have been linked to better emotional well-being and reduced anxiety [24]. Interventions like online meditation lessons have shown promise in reducing anxiety and improving socio-cultural adaptation for professional Indian immigrants [25]. Support from Indian family and community networks provides essential emotional and practical assistance and often serves as a key resource for discussing health concerns and seeking guidance [26,27].

While there is substantial literature that highlights sociocultural, economic, psychological, and environmental determinants of stress among South Asians, much of the work has been outdated, descriptive, and fragmented. Existing research observes isolated variables, such as work-family conflict [10], gender roles [12], or acculturative stress [20] without a unifying theoretical lens to understand how respective determinants interact and influence behavior change. Furthermore, acculturation theory or social support theory has been implied within the context of South Asian populations, but they fall short of encompassing the intricate, multi-level processes that drive both stress-inducing (e.g., avoidance, somatization) and stress-reducing behaviors (e.g., meditation, community engagement) [28,29].

Many of these models lack predictive power and practical application for intervention design, especially with contemporary health behavior change theories that have the potential to offer comprehensive frameworks for identifying modifiable determinants and mechanisms of change. A growing body of research exists on stress and relaxation techniques related to WLB. However, few studies employ behavioral theories to systematically examine the determinants of stress and relaxation, highlighting a critical gap in the literature. For instance, Nowrouzi et al. [30] reviewed the 50 most-cited studies on occupational stress and found that only 8% directly examined theories or models, with most research focusing on stress as either a predictor or an outcome variable, rather than explaining the mechanisms of behavior change. Similarly, Zaitouni et al. [31] conducted a bibliometric analysis of two decades of WLB literature and emphasized that while topics like gender roles and workplace flexibility are widely discussed, the field lacks a unified theoretical framework to explain how individuals manage stress and achieve balance. Ratnesh et al. [32] examined WLB across five Asian countries and discussed cultural and organizational influences, yet they did not apply behavioral theories to unpack how these factors translate into health-related behaviors. Even when psychological constructs such as coping strategies or emotional regulation are included [33], these are often evaluated in isolation, without grounding in comprehensive models of behavior change. The limited use of theories, when present, tends to be descriptive rather than explanatory or predictive, which restricts their utility in guiding intervention development.

It can be emphasized that the sociocultural determinants of stress among South Asian Americans are subject to scarcity of existing descriptive and atheoretical research. Prior studies have examined isolated factors such as gender roles, acculturation, and work–family conflict without an integrated behavioral framework, thereby establishing the need for a comprehensive theoretical model. This transition positions the novice Multi-Theory Model as the conceptual solution to prior fragmentation [34], linking the high stress burden in this population, the scarcity of theory-driven research on relaxation behaviors, and the suitability of MTM and the Perceived Stress Scale to address these gaps.

### 1.4. Multi-Theory Model of Health Behavior Change

Due to the complexity of stress and relaxation behavior, especially with South Asians in the U.S., a theory-driven approach is essential to identify the determinants and inform effective, culturally tailored interventions. The Multi-Theory Model (MTM) of health behavior change is a fourth-generation framework that cohesively synthesizes constructs from established behavioral theories to explain and influence both initiation and sustenance of health behaviors [34]. The initiation dimension focuses on initial factors that influence an individual’s behaviors. The key constructs are participatory dialogue (weighing advantages and disadvantages), behavioral confidence (self-efficacy), and changes in the physical environment. The sustenance dimension addresses the continuation of the behavior over time, emphasizing emotional transformation (channeling feelings toward goals), practice for change (ongoing self-regulation), and changes in the social environment (support systems that reinforce behavior) [35].

Several previous studies have examined the application of MTMs for various patient populations and diseases. For example, the MTM model has been applied to the screening of colorectal cancer prevention. In a theory-based model, it was used to assess screening behaviors and predictors of screening initiation and maintenance, as well as to identify barriers among adults aged 45–75 in the United States [36]. Similarly, this model has also been used to assess the initiation and sustenance behavior of smoking as well as in chronic diseases, for example, diabetes, to study behavior patterns to inform for targeted interventions and to improve the health status of elderly persons with diabetes [37,38]. Adaptation of MTM to specific target populations involves tailoring intervention strategies to address the unique barriers, facilitators, and cultural contexts relevant to those groups.

The Perceived Stress Scale (PSS) is one of the most widely used psychological instruments for measuring perception of stress across diverse populations [39]. This validated instrument utilizes the subjective experience of stress by asking individuals to reflect on their thoughts and feelings over the past month. It captures a balance between demands and perceived coping abilities, making it ideal for evaluating responses to chronic or situational stress [40]. Guided by the Multi-Theory Model of Health Behavior Change, the study hypothesized that higher participatory dialogue, behavioral confidence, and perceived changes in the physical environment would positively predict initiation of relaxation behavior, while higher emotional transformation, practice for change, and changes in the social environment would positively predict sustenance of relaxation behavior. Therefore, the primary aim of this study is to utilize the MTM and to examine the predictors of initiation and sustenance of relaxation behaviors among SAs residing in the United States together with the measurement of perceived stress amongst this diverse population. The secondary aim is to systematically identify and understand the relationship between perceived stress, MTM constructs, and relaxation practices in conjunction with the unique predictors of psychosocial, cultural, and environmental factors influencing these behaviors. The study will incorporate PSS to measure perceived stress levels, which will enable the assessment of how these subjective experiences relate to relaxation behavior and broader work–life balance dynamics within the SA population.

## 2. Materials and Methods

### 2.1. Study Design, Sample, and Study Participants

A cross-sectional study design is used in the current study, which is appropriate for testing theoretical structure, estimating relationships among constructs, and generating evidence for subsequent longitudinal or interventional research. This also aligns with established practice in MTM validation studies and behavioral theory testing. The sample size was calculated using G*Power 3.1.9.7 software (Heinrich-Heine-Universität Düsseldorf) [41], with an Alpha of 0.05, a power of 0.80, and an effect size of 0.10, resulting in a sample size of 270 (with an attrition of ~10% for a sample of 240 people). The data was gathered from March to April 2025. This survey focused on the conscious daily pursuit of relaxation behavior among individuals of South Asian origin or descent, including those from Afghanistan, Bangladesh, Bhutan, India, Maldives, Nepal, Pakistan, and Sri Lanka. Persons 18 years or older and identifying themselves as South Asian and/or through their parents or grandparents were eligible to participate. Eligible respondents were asked whether, within the past 24 h, they engaged in intentional relaxation activities other than sleep, and if so, to report the number of minutes spent on such practices.

### 2.2. Ethical Considerations

The University of Nevada, Las Vegas Institutional Review Board granted an exemption on 25 February 2025 for the study protocol number UNLV-2024-622. The consent form outlined the purpose, importance, and potential risks of the study. In addition, the consent form also clearly explained that participants could withdraw at any stage. Participation was voluntary, and only those who chose the “Agree” option were allowed to continue with the survey. No personal identifiers, including names or email addresses, were gathered.

### 2.3. Recruitment and Data Collection

Participant recruitment and data collection were conducted through QualtricsXM^®^ (Qualtrics, Provo, UT, USA), an online survey platform [42]. Quota sampling was employed to ensure a balanced representation of the target population. Eligible participants were invited to complete a web-based survey, which took approximately 10 min. All responses were collected anonymously through the Qualtrics^®^ system. Qualtrics^®^ recruited participants through targeted panels and specialized campaigns. To minimize self-selection bias, survey invitations are worded broadly. Based on the study’s eligibility requirements, screening questions were included at the start of the survey to ensure only qualified individuals participated. Those who met the criteria and completed the survey received compensation as outlined in the Qualtrics^®^ panel agreement.

### 2.4. Survey Instrument

The survey consisted of a 61-item questionnaire originally developed by the originator of the MTM theory [34] and subsequently refined through a two-stage expert panel review. Eight experts participated in a two-round process to assess the questionnaire’s face and content validity, and assessed the readability level (Supplementary Material File S1). The panel included two experts in health behavior research, mental health education, and instrument development; two in public health; two primary care internal medicine physicians who routinely care for patients with mental health disorders; and two South Asian mental health researchers. They were asked to evaluate each item for clarity, readability, and relevance. Based on their feedback, minor wording adjustments were made; however, no items were removed from the final instrument. All experts ultimately agreed that the content and face validity of each MTM subscale was adequate. The instrument demonstrated strong readability, with a Flesch Reading Ease score of 63.7 and a Flesch–Kincaid Grade Level of 5.9, indicating it was accessible and easy to understand for a general audience. In addition to MTM constructs, sociodemographic and behavioral factors were included as covariates because prior research indicates that stress experiences and coping behaviors are shaped by age, gender roles, employment conditions, socioeconomic status, acculturation, and baseline perceived stress. For example, extended work hours and limited sleep may reduce opportunity for relaxation practice, while higher perceived stress may increase motivation to seek coping strategies. Similarly, gendered caregiving expectations and occupational demands may influence behavioral confidence to initiate relaxation. Including these covariates allows examination of MTM mechanisms above and beyond contextual influence.

The final questionnaire started with four initial questions to determine the individual’s representation from their South Asian origin and their relaxation behavior in the last 24 h. The first two questions asked about the participant’s origin and/or descent from the South Asian population and age ≥ to 18 years. The second and third questions asked about the participant’s conscious engagement and time spent on any relaxation behavior. Participants were required to answer the first two screening questions before continuing with the rest of the survey.

This was followed by 10 items of the Perceived Stress Scale PSS-10 to measure stress-related experiences over the past month. These questions assess how often participants felt upset by unexpected events, unable to control important aspects of life, nervous or stressed, and whether they felt confident, in control, or that things were going their way. They also examined coping ability, control over irritations, feelings of being on top of tasks, anger from uncontrollable events, and whether problems seemed overwhelming. Responses were rated on a Likert Scale ranging from “Never” to “Very Often” score ranging from 0 to 4. Summative scores from all 10 items were obtained by reversing the scores on the four positive items, e.g., 0 = 4, 1 = 3, 2 = 2, etc., and then summing across all 10 items. Items 6, 7, 9, and 10 were the positively stated items. The possible range was 0–40. A high score on these four items indicated more stress.

Perceived stress was assessed as a covariate using the 10-item Perceived Stress Scale (PSS-10), a widely validated instrument used to measure subjective stress experiences. In this study, PSS scores were included as a covariate to control for baseline stress levels in regression models, rather than for psychometric evaluation.

There were 33 items assessing constructs from the Multi-Theory Model (MTM) related to the initiation and maintenance of relaxation behavior. Participatory dialogue (PD) was measured by assessing perceived advantages (five items) and perceived disadvantages (five items), each rated on a five-point scale from 0 = Never to 4 = Very often. Separate subscale scores ranged from 0 to 20, and the PD score was calculated by subtracting the perceived disadvantages score from the perceived advantages score, yielding a possible range from −20 to +20. Behavioral confidence was also measured with five items rated from 0 = Not at all sure to 4 = Completely sure, producing a score from 0 to 20. Changes in the physical environment (three items; e.g., “How sure are you that you will be able to have the necessary resources to practice relaxation?”), emotional transformation (three items; e.g., “How sure are you that you can motivate yourself to practice relaxation for 20 min daily?”), practice for change (three items; e.g., “How sure are you that you can be able to practice relaxation for 20 min daily if you encounter barriers?”), and changes in the social environment (three items; e.g., “How sure are you that you can get the help of a friend to support you with practicing relaxation for 20 min daily?”) were each assessed using the same 0–4 confidence scale, with possible subscale scores ranging from 0 to 12. Intent to initiate was measured with three items (e.g., “How likely is it that you will intend to practice relaxation for 20 min daily in the upcoming week?”), and intent to sustain was measured with three items (e.g., “How likely is it that you will consider practicing relaxation for 20 min daily from now on?”). Both the intent constructs used a five-point likelihood scale from 0 = Not at all likely to 4 = Completely likely, with total scores ranging from 0 to 12.

The remaining 16 items collected socio-demographic information, including gender, age, nationality, and time since living in the United States, residential state, employment status, academic level, tobacco and alcohol use, marital status, income, preexisting health conditions, and self-reported height and weight. The remaining 13 items collected socio-demographic information, including gender, age, race/ethnicity, religion, residential setting, employment status, academic level, tobacco and alcohol use, marital status, income, and preexisting health conditions.

### 2.5. Statistical Analysis

All data analyses were conducted using SPSS version 31.0 (IBM) [43] and lavaan package of R Statistical Software version 4.5.0 (R Core Team (2021) [44,45]). Descriptive statistics were reported as means and standard deviations for continuous variables, and as frequencies and percentages for categorical variables. Structural equation modeling (SEM) was first employed to confirm the construct validity and measurement integrity of MTM latent variables, ensuring that observed survey items accurately represented their intended theoretical constructs. Separate structural equation models were estimated for initiation and sustenance, consistent with the MTM framework, which conceptualizes these as distinct behavioral phases with unique latent constructs and indicator sets. Modeling them separately preserves theoretical fidelity, avoids unnecessary cross-loading of indicators, and allows phase-specific assessment of model fit and predictive pathways. SEM also evaluated overall model fit to verify that the hypothesized initiation and sustenance frameworks adequately reflected the observed data before advancing to predictive analyses. Subsequently, hierarchical regression was conducted to quantify the incremental explanatory contribution of each MTM construct while controlling for demographic and behavioral covariates, providing effect size estimates and practical interpretability for intervention planning. This sequential analytic strategy follows best practice in theory-driven behavioral research by confirming measurement validity, testing structural model fit, and estimating predictive contributions of theoretical constructs.

To assess internal consistency, Cronbach’s alpha coefficients were calculated for the overall scale and each subscale corresponding to the specific theoretical constructs. A threshold of 0.70 was set as the minimum acceptable alpha value, indicating sufficient reliability in measuring the intended constructs. To further assess reliability, one-dimensionality, and construct validity, structural equational modeling was performed in R, modeling each scale as a latent variable. Construct validity was evaluated by examining the correlations among the latent factors within a measurement model. Model fit was assessed using multiple fit indices: the Comparative Fit Index (CFI) and Tucker–Lewis Index (TLI), with values > 0.90 considered acceptable, and the Root Mean Square Error of Approximation (RMSEA) and Standardized Root Mean Square Residual (SRMR), with values < 0.08 indicating good fit. Effect sizes were interpreted using standard benchmarks: 0.10 (small), 0.30 (medium), and 0.50 (large).

The study examined two key outcomes: the intention to begin a relaxation behavior (initiation) and the likelihood of maintaining this behavior over time (sustenance). These outcomes were assessed using constructs from the Multi-Theory Model (MTM) of health behavior change. The independent variables included participatory dialogue, behavioral confidence, and changes in the physical environment for initiation, as well as emotional transformation, practice for change, and changes in the social environment for sustenance. In addition to that, participants who answered ‘Yes’ to engaging in intentional relaxation activity within the past 24 h were categorized as practicing conscious pursuit of relaxation behavior. Those who answered ‘No’ were categorized as not practicing exhibiting conscious pursuit of relaxation behavior. This grouping enabled separate modeling of initiation and sustenance predictors for individuals currently practicing versus not practicing relaxation behaviors. Covariates considered in the analysis included age, gender, nationality, health insurance, educational attainment, income level, marital status, smoking and alcohol use, PSS-10, diagnosis of mental illness, and daily frequency and duration of sleep and physical work including physical activity and domestic work. Participants reported average daily work and sleep hours to reflect behavior patterns, while relaxation time was assessed using a 24 h recall to capture recent engagement and reduce recall bias, providing a behavioral estimate despite potential day-to-day variability. Model adequacy was confirmed using established fit indices, including RMSEA, SRMR, CFI, and TLI.

Confirmatory factor analysis was initially conducted to test the construct validity of the MTM framework. Next, structural equation modeling was conducted to assess the overall fit and structural paths for initiation and sustenance. To demonstrate consistency and robustness, hierarchical multiple regression analyses were conducted to identify predictors of both the initiation and sustenance of stress relaxation behavior, following a stepwise modeling approach. For the initiation model, demographic and behavioral covariates were entered first (Model 1), followed by participatory dialogue (Model 2), behavioral confidence (Model 3), and changes in the physical environment (Model 4). A similar stepwise procedure was applied for the sustenance model, adding emotional transformation, practice for change, and changes in the social environment sequentially (Models 1–4). This approach allowed for assessing the incremental contribution of each MTM construct while controlling for other variables.

The regression models were evaluated to ensure they satisfied the assumptions of independence, linearity, normality, and homoscedasticity, confirmed using the Durbin–Watson statistic, partial regression plots, and the Shapiro–Wilk test. Multicollinearity was checked in the final models through the Variance Inflation Factor (VIF), with all analyses conducted at a 0.05 significance level. Statistical analyses were run to evaluate key regression assumptions, including independence of observations (Durbin–Watson), linearity (scatterplots and partial regression plots), homoscedasticity, multicollinearity, and normality (P–P and Q–Q plots). Levene’s test confirmed homogeneity of variance. The Durbin–Watson value was close to 2, indicating independent residuals. Visual inspection showed consistent variance, and tolerance values above 0.1 confirmed no multicollinearity concerns. No studentized deleted residuals exceeded ±3 standard deviations, leverage values remained below 0.2, and no Cook’s distance values exceeded 1. Normality of residuals was supported by the P–P Plot. Overall, all regression assumptions were adequately met.

## 3. Results

### 3.1. Sample Characteristics

The study sample consisted of 271 South Asian adults, with a mean age of 42.24 ± 14.64 years (*n* = 268) (Table 1). Participants reported an average of 25.85 ± 44.82 min of relaxation in the past 24 h (*n* = 257), alongside high daily work demands averaging 12.55 ± 4.5 h (*n* = 269) and 11.59 ± 4.1 h of sleep (*n* = 268). The gender distribution included 59.0% females, 39.5% males, and 0.4% identifying as other. Most participants were U.S. nationals (80.4%), with an average of 26.31 ± 17.52 years lived in the country. India represented the majority country of origin (52.9%), followed by Pakistan (11.4%), Nepal (7.4%), Bangladesh (6.3%), Sri Lanka (5.9%), Afghanistan (4.1%), Maldives (3.7%), and Bhutan (3.3%). Over half held a college or graduate degree (40.6%) or a postgraduate/professional degree (28% combined). Most were married (62.7%) and working for pay (68.6%). Employment sectors included IT (15.9%), healthcare (12.5%), retail (8.9%), and education (5.9%). A large majority had health insurance (89.3%). Smoking (21.4%) and alcohol use (46.5%) were reported by a subset of participants. Notably, 74.5% consciously engaged in relaxation behaviors, while 17% reported a mental illness diagnosis. Average stress levels measured by PSS-10 were 18.55 ± 5.5, with participants residing across all major U.S. regions.

Structural equation modeling is reported first to confirm the validity and theoretical integrity of the Multi-Theory Model followed by the hierarchical regression analyses which represent the primary analytic framework of this study and are used to evaluate the predictive strength, incremental contribution, and practical relevance of MTM constructs for initiation and sustenance of stress relaxation behaviors.

### 3.2. Internal Consistency and Construct Validity

The reliability analysis demonstrated strong internal consistency across all MTM constructs. Perceived Advantage (α = 0.84) and Perceived Disadvantage (α = 0.86) showed high reliability, while Behavioral Confidence achieved excellent consistency (α = 0.90). Changes in the Physical Environment (α = 0.87) and Emotional Transformation (α = 0.88) were similarly robust (Table 2). Practice for Change (α = 0.85) and Changes in the Social Environment (α = 0.81) reflected solid reliability. Both the Overall Initiation Scale (α = 0.90) and Overall Sustenance Scale (α = 0.91) demonstrated strong psychometric performance, with the Overall Scale achieving the highest reliability (α = 0.93), indicating excellent internal coherence.

**Figure 1:**
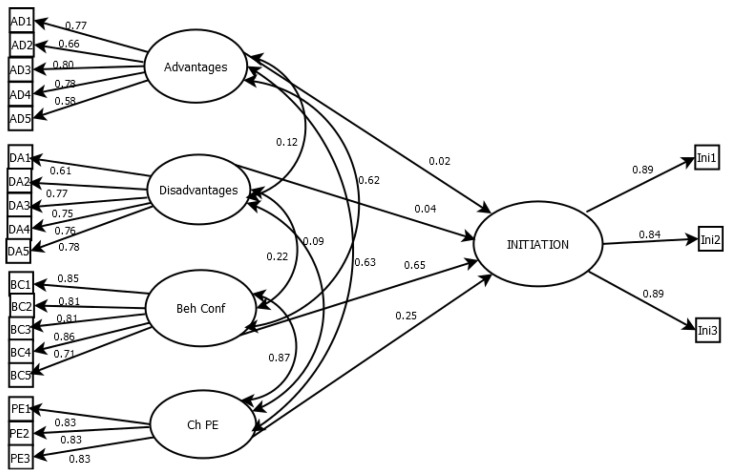
Path diagram of the structural equation model illustrating predictors of Initiation. Beh Conf = Behavioral Confidence; Ch PE = Changes in the Physical Environment.

The structural equation modeling (SEM) results for the Initiation model showed that the proposed framework fit the data well (Figure 1). Fit indices supported a strong model fit, explaining a robust model for the initiation (*x*^2^ [459] = 901.542 (*p* < 0.001), CFI = 0.953, TLI = 0.945, RMSEA = 0.060 [90% CI = 0.054–0.066], and SRMR = 0.05). All indices met recommended criteria, indicating that the hypothesized model aligned closely with the observed data.

Standardized factor loadings for the latent constructs of “Initiation” ranged from 0.58 to 0.86, showing that the measured items strongly represented their underlying variables. For the structural paths, “Initiation” was significantly predicted by Behavioral Confidence (β = 0.62, *p* < 0.001) and Changes in the Physical Environment (β = 0.63, *p* < 0.001). The model explained 61.6% of the variance in Initiation (R^2^ = 0.616), reflecting strong predictive capability. These results emphasize the importance of behavioral confidence and perceived benefits in shaping initiation behavior, consistent with theory suggesting that self-efficacy and perceived advantages are key drivers of action.

**Figure 2:**
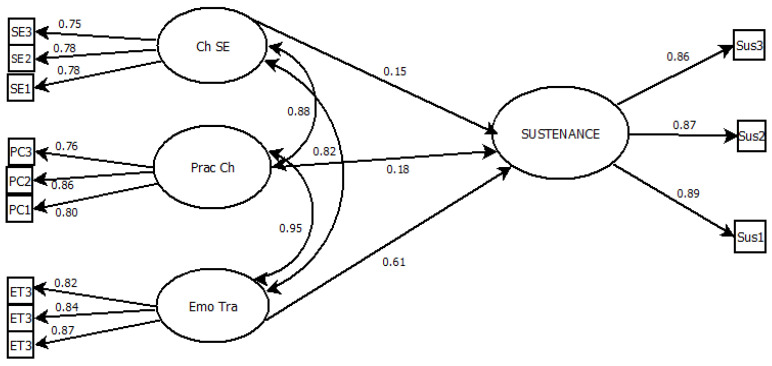
Path diagram of the structural equation model illustrating predictors of Sustenance. Emo Tran = Emotionsl Transformation; Pract Ch = Practice for Change; Ch SE = Changes in the Social Environment.

For the Sustenance model, SEM results indicated an excellent fit (Figure 2), with strong relevant indices also meeting the criteria that proposed indices closely aligned with the observed data (*x*^2^ [433] = 811.542 (*p* < 0.001), CFI = 0.922, TLI = 0.965, RMSEA = 0.056 [90% CI = 0.054–0.059], and SRMR = 0.07).

Standardized factor loadings for the latent constructs of “Sustenance” ranged from 0.75 to 0.87, showing that the measured items strongly represented their underlying variables. For the structural paths, Sustenance was significantly predicted by Emotional Transformation (β = 0.93, *p* < 0.001), Practice for Change (β = 0.94, *p* < 0.001), and Changes in the Social Environment (β = 0.75, *p* < 0.001).

### 3.3. Characteristics of MTM Constructs

Table 3 presents descriptive statistics for the Multi-Theory Model constructs among participants (*n* = 271). Both initiation (M ± 7.6, SD 3.3) and sustenance (M = 7.2, SD ± 3.3) displayed moderate mean scores, indicating similar levels of readiness to start and continue behavioral change. Perceived advantage (M = 13.07, SD ± 3.81) was markedly higher than perceived disadvantage (M = 9.78, SD ± 4.388), resulting in a positive participatory dialogue score (M = 3.29, SD ± 5.35), suggesting participants viewed benefits as outweighing barriers. Behavioral confidence was relatively strong (M = 16.5, SD ± 5.8), while changes in the physical environment (M = 8.4, SD ± 3.3) showed moderate environmental support. For sustenance, emotional transformation (M = 7.8, SD = 3.2), practice for change (M = 7.3, SD ± 3.1), and changes in the social environment (M = 8.1, SD ± 4.3) indicated moderate engagement in emotional coping, self-regulation practices, and supportive social influences essential for maintaining behavior change.

### 3.4. Correlations and Reliability Diagnostics of MTM Contracts

Table 4 shows strong, consistent relationships among core MTM constructs. Behavioral Confidence demonstrates the highest correlations across variables, strongly aligning with Emotional Transformation (*r* = 0.826), Changes in the Physical Environment (*r* = 0.773), Practice for Change (*r* = 0.790), and Social Environment (*r* = 0.706), highlighting its central role in behavior change. Physical Environment, Emotional Transformation, Practice for Change, and Social Environment are also highly interrelated (*r* = 0.625–0.811). In contrast, Participatory Dialogue shows weaker associations, significantly correlating only with Confidence, Physical Environment, and Emotional Transformation. Overall, sustenance constructs exhibit stronger interconnectedness than initiation constructs, reinforcing MTM’s theoretical structure.

### 3.5. Hierarchical Regression

Among the participants who were exhibiting conscious pursuit of stress relaxation behavior (*n* = 202), the hierarchical regression analyses demonstrated strong support for the Multi-Theory Model (MTM) in predicting both initiation and sustenance of conscious relaxation practices (Table 5). For initiation, Model 1 accounted for 32.0% of the variance, and the addition of participatory dialogue in Model 2 significantly increased explained variance to 44.3% (ΔR^2^ = 0.123, *p* < 0.05). Model 3, which included behavioral confidence, produced the largest increase (ΔR^2^ = 0.237, *p* < 0.001), raising the explained variance to 68.1%. The final model, incorporating changes in the physical environment, yielded the highest explanatory power with 69.8% of the variance explained (Adjusted R^2^ = 0.562), underscoring excellent model fit. Across these models, participatory dialogue and behavioral confidence remained significant predictors, with behavioral confidence emerging as the strongest contributor (β = 0.481–0.653). For sustenance, Model 1 explained 30.4% of the variance; however, the addition of emotional transformation in Model 2 led to a substantial improvement, nearly doubling the explained variance to 63.8% (ΔR^2^ = 0.334, *p* < 0.001). The inclusion of practice for change in Model 3 further increased the variance to 69.0% (ΔR^2^ = 0.052), and Model 4, adding changes in the social environment, produced the highest explanatory value at 70.4% variance explained (Adjusted R^2^ = 0.570). Emotional transformation was the strongest predictor of sustenance, followed by practice for change and social support. Collectively, these results highlight that initiation is predominantly driven by motivational and environmental readiness, whereas long-term maintenance depends heavily on emotional regulation, self-monitoring, and supportive social contexts.

Among the participants who were not exhibiting conscious pursuit of stress relaxation behavior (*n* = 68), the hierarchical regression analyses revealed that both the initiation and sustenance of relaxation behaviors were strongly influenced by MTM motivational constructs, though with different patterns than those observed among current practicing participants (Table 6). For initiation, Model 1 explained 88.3% of the variance, largely due to wide variability in baseline covariates, but the addition of participatory dialogue in Model 2 significantly increased the explained variance to 95.2% (ΔR^2^ = 0.069). Participatory dialogue emerged as the only significant MTM predictor, with a notably strong coefficient (β ≈ 0.95), underscoring that perceived advantages over disadvantages were the primary drivers of initial readiness to adopt relaxation practices. Behavioral confidence (Model 3) and changes in the physical environment (Model 4) contributed marginal increases in explained variance—raising the final model to having 97.2% variance explained (Adjusted R^2^ = 0.596)—but neither construct reached statistical significance, suggesting that participants who were not exhibiting a conscious pursuit of stress relaxation behavior did not rely on confidence or environmental support when contemplating behavior initiation. For sustenance, Model 1 accounted for 83.4% of variance, but the introduction of emotional transformation in Model 2 dramatically increased the explained variance to 94.5% (ΔR^2^ = 0.111, *p* < 0.01), with emotional transformation remaining the only significant MTM predictor across all models (β = 0.570–0.837). Subsequent additions of practice for change and changes in the social environment produced smaller increases, bringing the final model to 96.0% variance explained. Overall, these findings highlight that such participants who did not follow conscious pursuit of stress relaxation behavior rely heavily on motivational and emotional readiness—rather than confidence, environmental cues, or social support—both when considering initiation and when sustaining early attempts at relaxation behavior.

## 4. Discussion

This study examined predictors of the initiation and sustenance of conscious pursuit of relaxation behaviors among U.S.-based South Asian American adults, utilizing the Multi-Theory Model (MTM) of health behavior change as the guiding framework. The hierarchical regression models revealed that MTM constructs—including participatory dialogue, behavioral confidence, and changes in the physical environment—were significant predictors of initiating relaxation techniques among participants currently practicing relaxation behaviors. Similarly, emotional transformation, practice for change, and changes in the social environment were strong predictors of sustenance. These findings align conceptually and empirically with prior MTM-based studies demonstrating that individuals are more likely to adopt and maintain health behaviors when they perceive clear advantages, feel confident in performing the behavior, and have supportive environmental cues [46,47].

For individuals who were already practicing relaxation, Model 4 explained 69.8% of the variance in initiation and 70.4% of the variance in sustenance, indicating a robust explanatory power. Among MTM constructs, participatory dialogue emerged as a significant contributor (β = 0.194, *p* < 0.05), suggesting that individuals weigh advantages of relaxation—such as stress reduction and improved well-being—more heavily when making behavioral decisions, consistent with MTM’s emphasis on the advantages–disadvantages ratio.

Similarly, behavioral confidence significantly predicted initiation (β = 0.481, *p* < 0.001), affirming that confidence in one’s ability to schedule or engage in relaxation activities is critical for behavior change. This echoes previous research showing that MTM’s conceptualization of confidence, distinct from general self-efficacy, is a key driver of initiating new health behaviors such as physical activity or dietary changes [46,48].

For sustenance, the strong predictive contributions of emotional transformation (β = 0.395, *p* < 0.001) and practice for change (β = 0.307, *p* < 0.05) indicate that the ability to channel emotions into goals and establish consistent self-regulation strategies (e.g., reminders, tracking, coping strategies) is vital for maintaining ongoing relaxation routines. This is consistent with previous MTM applications showing emotional transformation as a stable predictor in sustaining behaviors such as screen-time reduction, meditation, mindfulness practice, and chronic disease management [49,50].

Among participants not currently practicing relaxation, MTM constructs were generally weaker predictors but still contributed meaningfully. Participatory dialogue predicted initiation (β ≈ 0.18–0.34), whereas emotional transformation remained significant for sustenance (β = 0.570, *p* < 0.001). This pattern suggests that individuals unfamiliar with a behavior may rely more on attitudinal and emotional readiness, whereas existing practitioners benefit more from self-regulatory and environmental supportive factors. The relatively lower model fit among non-practitioners also reflects the known challenge of establishing new behaviors among individuals facing cultural, occupational, or environmental constraints or who lack prior experience with stress-management practices.

Sociodemographic variables—including education, work–life balance, and alcohol use—also showed intermittent associations with MTM constructs. However, the consistency and strength of MTM variables highlight their centrality as behavioral change drivers across diverse population groups.

Overall, the findings strongly support the utility of the MTM framework in explaining and predicting conscious relaxation behavior among multiethnic South Asian populations in the U.S., reinforcing prior literature demonstrating MTM’s adaptability across cultural settings and health behaviors.

### Implications for Practice

The findings of this study offer several important implications for public health practice, particularly for interventions aimed at increasing the initiation and maintenance of relaxation behaviors among diverse South Asian adult populations. First, because behavioral confidence emerged as a strong predictor of initiation among current practitioners, interventions should prioritize skills training, guided demonstrations, and structured practice opportunities that enhance individuals’ confidence to incorporate relaxation into daily routines, consistent with MTM-based evidence showing that confidence is a primary driver of early behavior adoption [51].

The significance of participatory dialogue in both practitioners and non-practitioners indicates that programs must clearly communicate the advantages of relaxation—such as reduced stress and improved mental well-being—while addressing perceived barriers, which aligns with previous MTM applications emphasizing attitudinal clarity before behavior change occurs [47]. Additionally, the importance of environmental change for initiation among practitioners suggests that workplaces, community organizations, and health-care settings should create accessible physical spaces and cues supporting relaxation behaviors, a strategy consistent with environmental approaches in prior MTM-based interventions [48].

For sustaining the behavior, the strong influence of emotional transformation and practice for change highlights the necessity of integrating goal-setting, emotional reframing, self-monitoring tools, and ongoing reminders, as these self-regulatory strategies have been shown to promote long-term adherence in MTM-guided behavioral interventions [52]. Furthermore, since social environment significantly supported sustained practice among practitioners, interventions should incorporate peer encouragement, group-based relaxation activities, or culturally aligned social support structures that reinforce ongoing engagement. Together, these findings emphasize the need for culturally tailored, theory-driven approaches leveraging MTM constructs to effectively promote and sustain relaxation behaviors in South Asian communities living in the United States.

The Multi-Theory Model (MTM) of Health Behavior Change provides a valuable framework for understanding the complexities surrounding stress relaxation behaviors among South Asian Americans. This population faces unique stressors derived from acculturation and cultural expectations, notably impacting their mental health and coping mechanisms. Our study also indicates that South Asian Americans experience considerable acculturative stress, influenced by everyday racism and cultural dissonance related to their identities as immigrants or descendants of immigrants [6,53]. This stress can manifest in various mental health issues, emphasizing the importance of community-specific health interventions. The MTM posits that both emotional transformation and behavioral change are vital for developing sustainable health practices. For South Asian Americans, culturally relevant interventions that address behavioral confidence and environmental conditioning are essential for fostering long-term engagement in stress relaxation activities [47,54].

The sociocultural dynamics prevalent in South Asian American households, such as a strong emphasis on academic and professional success, can aggravate stress levels and establish internal barriers to help-seeking behaviors [19,54,55]. This internalization of societal expectations forms a unique psychological landscape that must be accounted for when designing stress-relief programs. The MTM can aid in tailoring these interventions, emphasizing the need for emotional support and social environments that encourage relaxation techniques such as mindfulness or community activities [56].

Existing literature highlights gendered experiences within these communities. Women often face compounded stress due to familial and cultural expectations, which can significantly influence their health behaviors [19,46]. Therefore, interventions tailored using the MTM should specifically consider gender dynamics, offering support that resonates with the lived experiences of South Asian women [6,54].

The MTM emphasizes the significance of understanding behavioral factors such as practice for change and emotional transformation. Emotional factors, particularly how stress affects cognitive functions and interpersonal relationships, should be central to any health education programs aimed at promoting relaxation behaviors [52,57]. South Asian Americans may benefit from community-based approaches that encourage both physical activity and cultural gatherings, which might relieve stress through social support mechanisms [46]. Although the present findings confirm the overall predictive utility of MTM constructs for relaxation behavior among South Asian adults, the practical meaning of these mechanisms likely varies across sociodemographic subgroups. For instance, behavioral confidence to initiate relaxation may depend on workplace flexibility, childcare availability, and family support among female professionals balancing employment and parenting responsibilities. In contrast, emotional transformation and practice for change among male unskilled or shift-based workers may be shaped more strongly by financial strain, job insecurity, peer networks, and community-based coping resources. Future research employing subgroup analyses, qualitative inquiry, and longitudinal designs is warranted to elucidate how MTM constructs operate within specific gender, occupational, and family contexts. Such work will facilitate development of culturally and contextually tailored interventions that leverage MTM mechanisms to promote sustainable stress-relaxation practices across diverse South Asian sub-populations.

This study has several limitations that should be considered when interpreting the findings. First, the cross-sectional design precludes causal inference or determination of temporal relationships between MTM constructs and relaxation behavior. Second, all data were self-reported, which may introduce recall and social desirability biases. Minutes of relaxation were assessed using a 24 h recall measure, which may not reflect typical long-term relaxation patterns due to daily variability in work schedules, family demands, or unexpected events. Work and sleep hours were reported as average daily estimates and may also be subject to reporting errors. Third, participants were recruited through an online survey panel, which may limit generalizability to South Asian Americans without internet access or those less inclined to participate in research surveys. Fourth, although a comprehensive set of covariates was included, unmeasured factors such as workplace policies, caregiving burden, or cultural coping practices may have influenced relaxation behavior. Finally, the study focused on one behavioral theory and one ethnic population, and findings may not generalize to other theoretical frameworks or cultural groups. Future research employing multi-day diary or ecological momentary assessment designs is warranted to capture habitual relaxation behavior more precisely.

## 5. Conclusions

This study provides strong evidence that the Multi-Theory Model (MTM) is an effective framework for understanding and predicting both the initiation and sustenance of conscious relaxation behaviors among South Asian adults in the United States. MTM constructs—particularly participatory dialogue, behavioral confidence, emotional transformation, and changes in physical and social environments—were central in predicting behavioral outcomes, even when accounting for extensive sociodemographic and lifestyle covariates. The findings emphasize that interventions aiming to promote stress-reducing relaxation behaviors should be multifaceted, culturally tailored, and grounded in behavioral theory. By leveraging MTM, public health practitioners can design effective, sustainable strategies to help diverse populations adopt and maintain behaviors that enhance mental health and overall well-being. Future research should incorporate longitudinal designs and qualitative exploration to enrich understanding of the cultural and structural factors influencing relaxation practices in immigrant communities. In conclusion, employing the Multi-Theory Model (MTM) for developing stress relaxation behavior intervention programs for South Asian Americans necessitates a comprehensive understanding of the community’s unique stressors and health behaviors. Integrating cultural insights into emotional support and social environments will enhance the efficacy of interventions aimed at alleviating stress within this underserved population.

## Figures and Tables

**Table 1:** Summary of Demographic Characteristics of the Sample (*n* = 271).

Variable	Characteristics	Mean ± SD	*n* (%)
Age * (*n* = 268)	-	42.24 ± 14.64	-
Minutes of Relaxation in past 24 h ** (*n* = 257)	-	25.85 ± 44.82	-
Work Hours/day (*n* = 269)	-	12.55 ± 4.5	
Sleep Hours/day (*n* = 268)	-	11.59 ± 4.1	
Gender (*n* = 268)	Male	-	107 (39.5)
	Female	-	160 (59.0)
	Others	-	1 (0.4)
Nationality	United States	-	218 (80.40)
	Other	-	47(17.3)
Years lived in the United States		26.31 ± 17.52	
Country of Origin	India	-	143 (52.9)
	Pakistan	-	31 (11.4)
	Nepal	-	20 (7.4)
	Sri Lanka	-	16 (5.9)
	Bangladesh	-	17 (6.3)
	Afghanistan	-	11 (4.1)
	Maldives	-	10 (3.7)
	Bhutan	-	9 (3.3)
Highest Level of Education	Some Schooling	-	6 (2.2)
	Completed HS or GED	-	40 (14.8)
	Some College	-	37 (13.7)
	Completed College/Graduate	-	110 (40.6)
	Post-Graduate Degree	-	58 (21.4)
	Professional Degree	-	18 (6.6)
Marital Status	Married	-	170 (62.7)
	Divorced	-	11 (4.1)
	Widowed	-	10 (3.7)
	Separated	-	4 (1.5)
	Never Married	-	59 (21.8)
	Civil Union/Domestic Partnership	-	8 (3.0)
	Member of Unmarried Couple	-	7 (2.6)
Work for Pay	Yes	-	186 (68.6)
	No	-	82 (30.3)
Employment Sector	Healthcare	-	34 (12.5)
	Information Technology	-	43 (15.9)
	Real Estate and Development	-	5 (1.8)
	Retail	-	24 (8.9)
	Education	-	16 (5.90)
	Government	-	4 (1.5)
	Other	-	60 (22.1)
Health Insurance	Yes	-	242 (89.3)
	No	-	19 (7.0)
Yearly Household Income	Less than $50,000	-	65 (24.0)
	$50,001 to $100,000	-	95 (35.1)
	$100,001 to $150,000	-	53 (19.6)
	$150,001 to $200,000	-	48 (17.7)
	None Reported	-	8 (3.0)
Smoking Status	Yes	-	58 (21.4)
	No	-	211 (77.9)
Frequency of Smoking	A few times a year	-	10 (3.7)
	At least once a month	-	13 (4.8)
	At least once a week	-	6 (2.2)
	At least once a day	-	29 (10.7)
Alcohol Consumption	Yes	-	126 (46.5)
	No	-	143 (52.8)
Frequency of Alcohol Consumption	Once in a while	-	33 (12.2)
	Sometimes	-	42 (15.5)
	At least once a week	-	36 (13.3)
	At least once a day	-	15 (5.5)
Conscious Pursuit of Relaxation	Yes	-	202 (74.5)
	No	-	68 (25.1)
Diagnosed with mental illness	Yes	-	46 (17.0)
	No	-	223 (82.3)
Work Life Balance	Excellent	-	41 (15.1)
	Very Good	-	90 (33.2)
	Good	-	87 (32.1)
	Fair	-	41 (15.1)
	Poor	-	10 (3.7)
Stress Level from Perceived Stress Scale (PSS-10)		18.55 ± 5.5	
Residence Region of the United States	Northeast	-	76 (28.0)
	Southwest	-	43 (15.9)
	West	-	64 (23.6)
	Southeast	-	43 (15.9)
	Midwest	-	44 (16.2)

Note. * Total sample size, ** Subjects reported Minutes of Relaxation in past 24 h.

**Table 2:** Reliability Estimation for the Initiation and Sustenance Scales and Subscales.

Scale	Cronbach’s Alpha (95% CI)
Perceived Advantage	0.84 (0.80, 0.87)
Perceived Disadvantage	0.86 (0.82, 0.89)
Behavioral Confidence	0.90 (0.87, 0.92)
Changes in the Physical Environment	0.87 (0.83, 0.90)
Overall Initiation Scale	0.90(0.88, 0.93)
Emotional Transformation	0.88 (0.84, 0.91)
Practice for Change	0.85 (0.80, 0.89)
Changes in the Social Environment	0.81 (0.75, 0.86)
Overall Sustenance Scale	0.91 (0.88, 0.93)
Overall Scale	0.93 (0.89, 0.97)

**Table 3:** Descriptive statistics of the multi-theory model of behavior change constructs (*n* = 270).

Constructs	Possible Score Range	Observed Score Range	Mean ± SD
Initiation	0–12	0–12	7.6 ± 3.3
Perceived Advantage (PA)	0–24	0–20	13.07 ± 3.81
Perceived Disadvantage (PDA)	0–24	0–20	9.78 ± 4.38
Participatory Dialogue (PA–PDA)	−24–+24	−9–+19	3.29 ± 5.35
Behavioral confidence	0–24	0–24	16.5 ± 5.8
Changes in the physical environment	0–12	3–12	8.4 ± 3.3
Sustenance	0–12	3–12	7.2 ± 3.3
Emotional transformation	0–12	0–12	7.8 ± 3.2
Practice for change	0–12	0–12	7.3 ± 3.1
Changes in the social environment	0–16	0–16	8.1 ± 4.3

**Table 4:** Pearson correlations between MTM constructs used in the study.

Variables	Participatory Dialogue	Behavioral Confidence	Changes in Physical Environment	Emotional Transformation	Practice for Change	Changes in Social Environment
Participatory Dialogue	1					
Behavioral Confidence	0.216 ** [0.098, 0.326]					
	*p* < 0.001					
Changes in the Physical Environment	0.321 ** [0.209, 0.424]	0.773 ** [0.719, 0.816]				
	*p* < 0.001	*p* < 0.001				
Emotional Transformation	0.281 ** [0.166, 0.387]	0.826 ** [0.783, 0.860]	0.793 ** [0.744, 0.833]			
	*p* < 0.001	*p* < 0.001	*p* < 0.001			
Practice for Change	0.103 [−0.017, 0.220]	0.790 ** [0.740, 0.831]	0.742 ** [0.682, 0.791]	0.811 ** [0.765, 0.848]		
	*p* = 0.092	*p* < 0.001	*p* < 0.001	*p* < 0.001		
Changes in the Social Environment	0.009 [−0.111, 0.128]	0.706 ** [0.640, 0.761]	0.625 ** [0.545, 0.692]	0.690 ** [0.621, 0.747]	0.753 ** [0.695, 0.800]	1
	*p* = 0.885	*p* < 0.001	*p* < 0.001	*p* < 0.001	*p* < 0.001	

** Correlation (*r*) is significant at *p* < 0.001.

**Table 5:** Hierarchical multiple regression to predict Initiation and Sustenance among those who are practicing conscious pursuit of relaxation behavior other than sleep (*n* = 202).

Variables		Model 1		Model 2		Model 3		Model 4	
		B	β	B	β	B	β	B	β
**Initiation**									
Constant		12.375 **		9.704 **		2.692		2.452	
Age		0.005	0.028	−0.006	−0.033	0.007	0.038	0.004	0.022
Minutes of Relaxation in past 24 h		−0.008	−0.085	−0.007	−0.070	0.001	0.007	0.003	0.036
Work Hours/day		−0.012	−0.018	−0.013	−0.018	−0.016	−0.023	−0.017	−0.024
Sleep Hours/day		0.077	0.061	0.079	0.063	0.083	0.066	0.029	0.023
Gender (Ref: Female)	Male	0.537	0.096	0.300	0.053	0.543	0.097	0.425	0.076
Nationality (Ref: United States)		−0.789	−0.102	−0.778	−0.101	−0.099	−0.013	−0.077	−0.010
Years lived in United States		−0.005	−0.030	−0.008	−0.048	0.002	0.011	0.001	0.008
Country of Origin (Ref: India)	Pakistan	−0.677	−0.089	−0.286	−0.038	−0.417	−0.055	−0.526	−0.069
	Nepal	−0.262	−0.029	0.231	0.026	−0.231	−0.026	−0.378	−0.037
	Sri Lanka	−1.323	−0.140	−1.613	−0.171	−1.185	−0.125	−1.083	−0.115
	Bangladesh	−0.138	−0.015	−0.176	−0.019	−0.470	−0.050	−0.737	−0.078
	Afghanistan	−1.068	−0.113	−0.347	−0.037	−0.564	−0.060	−0.506	−0.054
	Maldives	−2.086	−0.203	−1.030	−0.100	−0.664	−0.065	−0.378	−0.37
	Bhutan	0.107	0.009	−0.005	0.000	1.072	0.086	0.935	0.075
Highest Level of Education (Ref: Completed College/Graduate)	Some Schooling	−1.495	−0.087	−2.291	−0.133	−0.985	−0.057	−0.838	−0.048
	Completed HS or GED	−0.872	−0.110	−0.890	−0.112	−0.104	−0.013	−0.030	−0.004
	Some College	−0.900	−0.102	−1.212	−0.137	−0.942	−0.107	−0.839	−0.095
	Post-Graduate Degree	−0.408	−0.061	−0.229	−0.034	−0.610	−0.090	−0.613	−0.091
	Professional Degree	−1.495	−0.087	−2.179	−0.192	−1.080	−0.095	−0.763	−0.067
Marital Status (Ref: Married)	Divorced	1.372	0.100	1.624	0.118	0.735	0.053	0.454	0.033
	Widowed	1.626	0.111	1.276	0.087	0.850	0.058	0.928	0.063
	Separated	−0.567	−0.021	−0.075	−0.003	1.784	0.066	1.307	0.048
	Never Married	0.375	0.056	0.428	0.064	0.421	0.063	0.211	0.032
	Civil Union /Domestic Partnership	0.826	0.052	0.952	0.060	1.663	0.105	1.344	0.085
	Member of Unmarried Couple	−0.386	−0.020	−0.644	−0.033	0.735	0.053	−0.682	−0.035
Work for Pay (Ref: Yes)		−0.076	−0.012	0.101	0.016	−0.007	−0.001	−0.049	−0.008
Employment Sector (Ref: Other)	Healthcare	0.371	0.045	0.238	0.029	0.236	0.028	0.358	0.043
	Information Technology	−0.057 *	−0.046	−0.069	−0.055	−0.034	−0.027	−0.061	−0.048
	Real Estate and Development	2.034	0.106	1.912	0.099	0.410	0.021	0.557	0.029
	Retail	0.006	0.001	−0.061	−0.006	0.177	0.018	0.376	0.039
	Education	−1.201	−1.01	−0.456	−0.038	−0.843	−0.071	−0.652	−0.055
	Government	2.211	0.115	2.348	0.122	0.595	0.031	0.474	0.025
Health Insurance (Ref: Yes)		0.229	0.020	0.238	0.029	−0.538	−0.047	−0.630	−0.055
Yearly Household Income (Ref: $50,001 to $100,000)	Less than $50,000	−0.212	−0.030	0.077	0.011	−0.151	−0.022	0.051	0.007
	$100,001 to $150,000	0.317	0.046	0.211	0.030	−0.218	−0.032	−0.223	−0.032
	$150,001 to $200,000	−0.491	−0.071	−0.384	−0.055	−0.244	−0.035	−0.138	−0.020
	None Reported	−1.409	−0.073	−0.659	−0.034	−0.533	−0.028	−0.522	−0.027
Smoking Status (Ref: No)		0.964	0.145	0.740	0.112	−0.337	−0.051	−0.041	−0.006
Frequency of Smoking (Ref: Once a day)	A few times a year	−1.102	−0.057	0.856	0.044	1.400	0.073	0.905	0.047
	At least once a month	−1.324	−0.107	−0.592	−0.048	−0.912	−0.074	−0.945	−0.076
	At least once a week	0.264	0.017	−0.250	−0.016	0.432	0.027	0.262	0.017
Alcohol Consumption (Ref: No)		−0.631	−0.113	−0.601	−0.108	−0.333	−0.060	−0.273	−0.049
Frequency of Alcohol Consumption (Ref: Sometimes)	Once in a while	0.567	0.065	0.352	0.041	0.076	0.009	−0.032	−0.004
	At least once a week	−1.376	−0.171	−1.121	−0.139	−0.541	−0.067	−0.772	−0.096
	At least once a day	2.099	0.191	2.146	0.196	0.717	0.065	0.387	0.035
Diagnosed with mental illness (Ref: No)		−0.390	−0.054	0.166	0.023	0.183	0.025	0.266	0.037
Work Life Balance (Ref: Very Good)	Excellent	0.122	0.016	0.593	0.078	0.040	0.005	0.133	0.018
	Good	−1.315	−0.225	−0.910	−0.156	−0.318	−0.054	−0.273	−0.047
	Fair	−1.082	−0.134	−1.581	−0.196	0.009	0.001	−0.040	−0.005
	Poor	4.776	0.215	4.158	0.187	1.747	0.079	1.875	0.084
Stress Level from Perceived Stress Scale (PSS-10)		−0.043	−0.078	0.062	0.113	−0.008	−0.015	−0.016	−0.030
Residence of region of United States	Southwest	0.285	0.038	−0.180	−0.024	0.057	0.007	0.011	0.001
	West	0.335	0.048	0.354	0.051	0.089	0.013	0.196	0.028
	Southeast	−0.387	−0.054	−0.546	−0.076	−0.251	−0.035	−0.316	−0.044
	Midwest	1.022	0.136	0.930	0.124	0.647	0.086	0.518	0.069
Participatory Dialogue				0.247 **	0.485	0.122 **	0.239 **	0.099 *	0.194 *
Behavioral Confidence						0.431 **	0.653 **	0.318 **	0.481 **
Changes in the Physical Environment								0.262 **	0.242 **
R^2^		0.320		0.443		0.681		0.698	
F		1.121		1.849 *		4.821 **		5.110 **	
ΔR^2^		0.320		0.123		0.237		0.018	
**Sustenance**									
Constant		12.151 *		3.587 *		2.891		2.540	
Age		0.022	0.119	0.021	0.111	0.022	0.118	0.030	0.164
Minutes of Relaxation in past 24 h		−0.018	−0.186	−0.009	−0.098	−0.008	−0.082	−0.008	−0.088
Work Hours/day		−0.055	−0.081	−0.031	−0.046	−0.041	−0.061	−0.056	−0.082
Sleep Hours/day		0.111	0.090	0.006	0.005	0.012	0.010	0.027	0.022
Gender (Ref: Female)	Male	0.006	0.001	−0.198	−0.036	−0.214	−0.039	−0.238	−0.043
Nationality (Ref: United States)		−1.266	−0.168	−1.006	−0.134	−0.670	−0.089	−0.641	−0.085
Years lived in United States		−0.031	−0.198	−0.026	−0.167	−0.021	−0.135	−0.024	−0.151
Country of Origin (Ref: India)	Pakistan	−0.535	−0.072	−0.490	−0.066	−0.468	−0.063	−0.565	−0.076
	Nepal	−0.122	−0.014	0.016	0.002	−0.238	−0.027	−0.368	−0.042
	Sri Lanka	−0.860	−0.093	−0.009	−0.098	−0.673	−0.073	−0.679	−0.074
	Bangladesh	0.387	0.042	−0.231	−0.025	−0.112	−0.012	−0.178	−0.019
	Afghanistan	−0.570	−0.062	0.127	0.014	0.272	0.029	0.033	0.004
	Maldives	−0.910	−0.091	−0.196	−0.020	0.226	0.023	0.289	0.029
	Bhutan	−0.994	−0.082	−0.534	−0.044	−0.355	−0.029	−0.281	−0.023
Highest Level of Education (Ref: Completed College/Graduate)	Some Schooling	−2.611	−0.155	−0.547	−0.032	−0.695	−0.041	−0.589	−0.035
	Completed HS or GED	−1.039	−0.134	−0.774	−0.100	−0.781	−0.101	−0.810	−0.105
	Some College	−0.798	−0.093	−0.516	−0.060	−1.011	−0.117	−0.985	−0.114
	Post-Graduate Degree	−0.288	−0.044	−0.757	−0.115	−0.839	−0.128	−0.991	−0.151
	Professional Degree	−0.686	−0.062	0.874	0.079	0.656	0.059	0.501	0.045
Marital Status (Ref: Married)	Divorced	1.494	0.111	0.779	0.058	0.778	0.058	0.481	0.036
	Widowed	0.328	0.023	−0.215	−0.015	−0.832	−0.058	−0.783	−0.055
	Separated	−1.770	−0.067	0.565	0.021	0.871	0.033	0.948	0.036
	Never Married	0.292	0.045	0.097	0.015	0.186	0.029	0.247	0.038
	Civil Union /Domestic Partnership	1.293	0.084	1.479	0.096	1.468	0.095	1.331	0.086
	Member of Unmarried Couple	0.060	0.003	−0.225	−0.012	−0.559	−0.030	−0.427	−0.023
Work for Pay (Ref: Yes)		−0.073	−0.012	−0.258	−0.042	−0.167	−0.027	−0.414	−0.067
Employment Sector (Ref: Other)	Healthcare	−0.003	0.000	−0.286	−0.035	−0.046	−0.006	−0.052	−0.006
	Information Technology	0.009	0.007	−0.014	−0.011	−0.061	−0.049	−0.038	−0.030
	Real Estate and Development	−0.297	−0.016	−1.475	−0.078	−1.397	−0.074	−1.557	−0.083
	Retail	0.074	0.008	−0.184	−0.019	0.382	0.040	0.395	0.042
	Education	−0.356	−0.031	−0.333	−0.029	−0.179	−0.015	−0.362	−0.031
	Government	3.301	0.175	1.004	0.053	0.897	0.048	0.945	0.050
Health Insurance (Ref: Yes)		1.282	0.115	0.132	0.012	0.372	0.034	0.591	0.053
Yearly Household Income (Ref: $50,001 to $100,000)	Less than $50,000	0.034	0.005	0.475	0.069	0.443	0.065	0.584	0.085
	$100,001 to $150,000	0.522	0.077	0.526	0.078	0.436	0.064	0.448	0.066
	$150,001 to $200,000	0.101	0.015	0.550	0.081	0.630	0.093	0.584	0.086
	None Reported	−1.144	−0.061	−0.866	−0.046	−0.287	−0.015	−0.333	−0.018
Smoking Status (Ref: No)		0.805	0.124	0.032	0.005	−0.018	−0.003	0.041	0.006
Frequency of Smoking (Ref: Once a day)	A few times a year	−1.175	−0.062	−0.521	−0.028	−0.380	−0.020	−0.642	−0.034
	At least once a month	0.522	0.043	0.690	0.057	0.415	0.034	0.223	0.018
	At least once a week	0.102	0.007	0.119	0.008	0.297	0.019	0.398	0.026
Alcohol Consumption (Ref: No)		−0.298	−0.055	−0.387	−0.071	−0.586	−0.108	−0.459	−0.084
Frequency of Alcohol Consumption (Ref: Sometimes)	Once in a while	0.877	0.104	1.559	0.184	1.749	0.207	1.581	0.187
	At least once a week	−1.059	−0.135	−0.429	−0.054	−0.013	−0.002	−0.056	−0.007
	At least once a day	2.124	0.198	0.803	0.075	0.745	0.070	0.431	0.040
Diagnosed with mental illness (Ref: No)		−0.488	−0.069	−0.207	−0.029	−0.412	−0.058	−0.429	−0.061
Work Life Balance (Ref: Very Good)	Excellent	0.187	0.025	−0.625	−0.084	−0.803	−0.108	−0.535	−0.072
	Good	−1.359	−0.239	−0.659	−0.116	−0.478	−0.084	−0.414	−0.073
	Fair	−1.028	−0.131	0.318	0.040	0.252	0.032	0.455	0.058
	Poor	3.816	0.176	0.287	0.013	0.876	0.040	0.681	0.031
Stress Level from Perceived Stress Scale (PSS-10)		−0.040	−0.074	−0.017	−0.032	−0.032	−0.060	−0.044	−0.083
Residence region of the United States	Southwest	0.566	0.076	0.970	0.131	0.924	0.125	0.925	0.125
	West	0.469	0.069	0.390	0.058	0.435	0.064	0.377	0.056
	Southeast	−0.033	−0.005	0.576	0.082	0.684	0.097	0.448	0.063
	Midwest	0.954	0.130	0.360	0.049	0.230	0.031	0.418	0.057
Emotional Transformation				0.773 **	0.747 **	0.455 **	0.440 **	0.409 **	0.395 **
Practice for Change						0.409 **	0.424 **	0.296 *	0.307 *
Changes in the Social Environment								0.198 *	0.210 *
R^2^		0.304		0.638		0.690		0.704	
F		1.041		4.094 **		5.030 **		5.243 **	
ΔR^2^		0.304		0.334		0.052		0.014	

For Initiation: * *p* < 0.05; ** *p* < 0.001; Adjusted R^2^ for Model 4 = 0.562. For Sustenance: * *p* < 0.05; ** *p* < 0.001; Adjusted R^2^ for Model 4 = 0.570.

**Table 6:** Hierarchical multiple regression to predict Initiation and Sustenance among those who are not practicing conscious pursuit of relaxation behavior other than sleep (*n* = 68).

Variables		Model 1		Model 2		Model 3		Model 4	
		B	β	B	β	B	β	B	β
**Initiation**									
Constant		5.248		10.965		3.362		−0.527	
Age		0.082	0.362	−0.066	−0.291	0.008	0.036	0.039	0.171
Minutes of Relaxation in past 24 h		−0.040	−0.910	−0.038	−0.873	−0.027	−0.614	−0.033	−0.742
Work Hours/day		0.147	0.175	−0.085	−0.101	0.030	0.035	0.075	0.089
Sleep Hours/day		0.297	0.177	0.356	0.213	0.163	0.097	0.150	0.089
Gender (Ref: Female)	Male	−2.041	−0.279	−3.668	−0.502	−1.081	−0.148	−0.755	−0.103
Nationality (Ref: United States)		−1.592	−0.199	−2.386	−0.298	−0.127	−0.016	−0.864	−0.108
Years lived in United States		−0.005	−0.030	−0.197	−1.031	−0.147	−0.766	−0.139	−0.728
Country of Origin (Ref: India)	Pakistan	5.655	0.463	−0.483	−0.040	1.573	0.129	2.650	0.217
	Nepal	8.359	0.743	9.957	0.885	5.984	0.532	4.453	0.396
	Sri Lanka	5.556	0.411	6.325	0.468	3.871	0.286	5.639	0.417
	Bangladesh	6.034	0.494	8.005	0.655	4.050	0.332	3.811	0.312
	Afghanistan	−4.981	−0.265	−1.837	−0.098	−2.265	−0.121	−4.487	−0.239
	Maldives	−1.518	−0.124	3.937	0.322	−0.072	−0.006	−1.038	−0.085
	Bhutan	3.472	0.185	9.628	0.512	2.634	0.140	2.001	0.107
Highest Level of Education (Ref: Completed College/Graduate)	Some Schooling	-	-	-	-	-	-	-	-
	Completed HS or GED	−4.890	−0.465	−5.428	−0.516	−1.520	−0.144	−4.005	−0.381
	Some College	−0.767	−0.085	1.273	0.140	−0.071	−0.008	−1.643	−0.181
	Post-Graduate Degree	−3.429	−0.405	−0.450	−0.053	−0.918	−0.108	−1.282	−0.151
	Professional Degree	−13.803	−0.735	−7.608	−0.405	−4.541	−0.242	−4.977	−0.265
Marital Status (Ref: Married)	Divorced	3.962	0.256	11.710	0.756	5.812	0.375	3.455	0.223
	Widowed	−4.509	−0.171	3.646	0.138	−0.245	−0.009	−3.755	−0.143
	Separated	-	-	-	-	-	-	-	
	Never Married	−1.391	−0.174	−3.625	−0.452	−0.649	−0.081	0.239	0.030
	Civil Union /Domestic Partnership	-	-	-	-	-	-	-	-
	Member of Unmarried Couple	4.419	0.235	−1.347	−0.072	0.765	0.041	2.788	0.148
Work for Pay (Ref: Yes)		−0.178	−0.025	0.367	0.052	−0.047	−0.007	−0.577	−0.082
Employment Sector (Ref: Other)	Healthcare	0.828	0.079	1.469	0.140	0.079	0.007	0.869	0.083
	Information Technology	−0.055 *	−0.043	−0.064	−0.050	−0.037	−0.021	−0.062	−0.046
	Real Estate and Development	4.835	0.184	1.323	0.050	4.720	0.179	5.847	0.222
	Retail	−4.593	−0.376	−5.181	−0.424	−4.254	−0.348	−1.840	−0.151
	Education	10.281	0.547	9.006	0.479	4.852	0.258	3.984	0.212
	Government	-	-	-	-	-	-	-	-
Health Insurance (Ref: Yes)		4.299	0.352	1.465	0.120	2.777	0.227	3.530	0.289
Yearly Household Income (Ref: $50,001 to $ 100,000)	Less than $50,000	8.589	1.216	6.763	0.957	4.555	0.645	5.447	0.771
	$100,001 to $150,000	1.600	0.169	0.784	0.083	1.236	0.131	1.737	0.183
	$150,001 to $200,000	8.800	0.836	8.392	0.797	5.680	0.540	5.969	0.567
	None Reported	10.425	0.673	6.629	0.428	7.354	0.475	8.964	0.579
Smoking Status (Ref: No)		8.133	0.722	2.552	0.227	0.742	0.066	3.512	0.312
Frequency of Smoking (Ref: Once a day)	A few times a year	−10.475	−0.398	−0.599	−0.023	−2.345	−0.089	−7.655	−0.291
	At least once a month	−30.304	−1.151	−22.540	−0.856	−9.516	−0.361	−11.811	−0.448
	At least once a week	-	-	-	-	-	-	-	-
Alcohol Consumption (Ref: No)		5.780	0.818	3.187	0.451	4.350	0.616	5.512	0.780
Frequency of Alcohol Consumption (Ref: Sometimes)	Once in a while	−4.469	−0.472	−1.755	−0.185	−3.130	−0.331	−5.098	−0.538
	At least once a week	−1.439	−0.145	−1.527	−0.154	−1.862	−0.187	−3.953	−0.398
	At least once a day	25.280	0.960	20.973	0.796	15.794	0.600	17.604	0.668
Diagnosed with mental illness (Ref: No)		1.671	0.089	1.246	0.066	4.312	0.229	3.981	0.212
Work Life Balance (Ref: Very Good)	Excellent	−8.042	−0.764	−5.375	−0.511	−2.383	−0.226	−2.439	−0.232
	Good	−1.061	−0.132	0.112	0.014	1.035	0.129	0.894	0.112
	Fair	−9.905	−0.996	−11.186	−1.125	−4.998	−0.503	−4.702	−0.473
	Poor	−6.565	−0.583	0.343	0.030	−1.912	−0.170	−2.439	−0.232
Stress Level from Perceived Stress Scale (PSS-10)		−0.063	−0.124	0.033	0.064	0.001	0.001	0.029	0.058
Residence Region of United States	Southwest	4.001	0.423	−0.180	−0.024	−0.540	−0.057	0.511	0.054
	West	2.330	0.304	0.354	0.051	0.916	0.119	1.055	0.138
	Southeast	−0.593	−0.056	−0.546	−0.076	−1.870	−0.178	−1.618	−0.154
	Midwest	4.805	0.529	0.930	0.124	2.758	0.304	3.024	0.333
Participatory Dialogue				0.619 *	0.955 *	0.222	0.343	0.119	0.184
Behavioral Confidence						0.240	0.405	0.116	0.196
Changes in the Physical Environment								0.395	0.410
R^2^		0.883		0.952		0.967		0.972	
F		1.055		2.346		2.855		2.588	
ΔR^2^		0.883		0.069		0.015		0.004	
**Sustenance**									
Constant		10.536		−2.410		−3.645		−6.979	
Age		0.023	0.095	0.035	.144	0.054	0.220	0.080	0.329
Minutes of Relaxation in past 24 h		−0.058	−1.231	−0.030	−0.629	−0.028	−0.596	−0.007	−0.140
Work Hours/day		0.029	0.478	−0.103	−0.115	−0.142	−0.158	−0.158	−0.176
Sleep Hours/day		0.331	0.185	0.035	0.020	0.021	0.012	−0.026	−0.014
Gender (Ref: Female)	Male	−3.879	−0.497	−0.639	−0.082	−0.001	0.000	1.379	0.177
Nationality (Ref: United States)		−2.640	−0.308	−0.389	−0.045	−0.001	0.000	−0.983	−0.115
Years lived in United States		−0.183	−0.896	−0.058	−0.284	−0.045	−0.222	−0.077	−0.379
Country of Origin (Ref: India)	Pakistan	5.238	0.401	2.142	0.164	3.879	0.297	1.386	0.106
	Nepal	10.003	0.831	3.220	0.268	2.129	0.177	1.313	0.109
	Sri Lanka	2.885	0.199	3.772	0.261	3.976	0.275	4.437	0.307
	Bangladesh	5.938	0.455	1.181	0.090	0.066	0.005	−0.482	−0.037
	Afghanistan	−2.569	−0.128	−0.709	−0.035	−0.518	−0.026	−4.560	−0.227
	Maldives	−0.733	−0.056	0.625	0.048	0.860	0.066	0.194	0.015
	Bhutan	7.515	0.374	0.272	0.014	−1.312	−0.065	−3.847	−0.192
Highest Level of Education (Ref: Completed College/Graduate)	Some Schooling	-	-	-	-	-	-	-	-
	Completed HS or GED	−4.305	−0.383	−2.926	−0.260	−2.723	−0.242	−3.170	−0.282
	Some College	0.465	0.048	−1.167	−0.120	−1.674	−0.173	−1.868	−0.193
	Post-Graduate Degree	−0.252	−0.028	0.532	0.059	0.746	0.082	0.645	0.071
	Professional Degree	−14.816	−0.738	−5.585	−0.278	−3.869	−0.193	−1.882	−0.094
Marital Status (Ref: Married)	Divorced	1.700	0.103	−1.658	−0.100	−1.706	−0.103	−0.455	−0.027
	Widowed	−4.422	−0.157	−3.823	−0.136	−3.655	−0.130	−2.155	−0.077
	Separated	-	-	-	-	-	-	-	-
	Never Married	−2.875	−0.336	0.426	0.050	1.416	0.165	1.033	0.121
	Civil Union /Domestic Partnership	-	-	-	-	-	-	-	-
	Member of Unmarried Couple	3.540	0.176	4.412	0.220	5.850	0.291	2.999	0.149
Work for Pay (Ref: Yes)		0.395	0.053	−0.848	−0.113	−1.738	−0.232	−2.520	−0.336
Employment Sector (Ref: Other)	Healthcare	−0.819	−0.073	1.116	0.099	0.889	0.079	1.029	0.091
	Information Technology	0.007	0.005	−0.012	−0.014	−0.061	−0.049	−0.036	−0.033
	Real Estate and Development	6.234	0.221	6.145	0.218	8.425	0.299	1.311	0.047
	Retail	−6.391	−0.490	−3.022	−0.231	−2.042	−0.156	−1.520	−0.116
	Education	10.128	0.504	3.739	0.186	1.987	0.099	−2.116	−0.105
	Government	-	-	-	-	-	-	-	-
Health Insurance (Ref: Yes)		2.323	0.178	1.418	0.109	1.487	0.114	2.558	0.196
Yearly Household Income (Ref: $50,001 to $ 100,000)	Less than $50,000	7.869	1.042	4.544	0.602	3.865	0.512	4.487	0.594
	$100,001 to $150,000	0.323	0.032	1.344	0.133	1.484	0.147	3.305	0.327
	$150,001 to $200,000	3.572	0.318	1.115	0.099	−0.386	−0.034	1.694	0.151
	None Reported	5.545	0.335	5.080	0.307	6.518	0.394	6.058	0.366
Smoking Status (Ref: No)		7.306	0.607	1.959	0.163	0.369	0.031	0.599	0.050
Frequency of Smoking (Ref: Once a day)	A few times a year	−10.046	−9.244	−0.328	0.044	−10.026	−0.356	−12.310	−0.437
	At least once a month	−30.710	−7.825	−0.278	−0.048	−1.709	−0.061	2.352	0.084
	At least once a week	-	-	-	-	-	-	-	-
Alcohol Consumption (Ref: No)		2.669	0.353	−0.458	−0.061	−0.008	−0.001	0.869	0.115
Frequency of Alcohol Consumption (Ref: Sometimes)	Once in a while	−1.554	−0.154	−2.828	−0.279	−3.257	−0.322	−5.070	−0.501
	At least once a week	2.632	0.248	0.753	0.071	−0.101	−0.010	−1.879	−0.177
	At least once a day	36.491	1.296	15.948	0.566	16.845	0.598	3.019	0.107
Diagnosed with mental illness (Ref: No)		0.793	0.040	1.207	0.060	2.074	0.103	0.197	0.010
Work Life Balance (Ref: Very Good)	Excellent	−9.595	−0.853	−1.592	−0.142	−0.169	−0.015	0.570	0.051
	Good	−2.547	−0.297	−0.452	−0.053	0.036	0.004	1.026	0.120
	Fair	−11.775	−1.108	−4.342	−0.409	−2.826	−0.266	−1.349	−0.127
	Poor	−5.072	−0.422	−4.917	−0.409	−5.848	−0.486	−2.097	−0.174
Stress Level from Perceived Stress Scale (PSS-10)		−0.112	−0.207	0.117	0.217	0.092	0.170	0.126	0.232
Residence Region of the United States	Southwest	1.806	0.220	2.646	0.261	3.262	0.322	1.892	0.187
	West	0.959	0.085	1.108	0.135	0.732	0.089	2.848	0.347
	Southeast	3.758	0.387	1.025	0.091	0.593	0.053	1.383	0.123
	Midwest	1.806	0.220	2.864	0.295	2.802	0.289	2.698	0.278
Emotional Transformation				0.923 *	0.837 *	0.649 *	0.589 *	0.628 *	0.570 *
Practice for Change						0.372	0.357	−0.075	−0.072
Changes in the Social								0.638	0.622 *
R^2^		0.834		0.945		0.951		0.960	
F		0.702		2.010		1.847		1.816	
ΔR^2^		0.834		0.111		0.006		0.010	

For Initiation: * *p* < 0.05; Adjusted R^2^ for Model 4 = 0.875. For Sustenance: * *p* < 0.05; Adjusted R^2^ for Model 4 = 0.824.

## Data Availability

Due to ethical restrictions, the data obtained and analyzed during the study are not publicly available and can be obtained from the corresponding author upon reasonable request.

## Supplementary Materials

The following supporting information can be downloaded at: https://www.mdpi.com/article/10.3390/ijerph23020253/s1, File S1: Survey Questionnaire.

## Abbreviations

The following abbreviations are used in this manuscript:

MTM	Multi-Theory Model
SEM	Structural equation modeling
CFI	Comparative Fit Index
TLI	Tucker–Lewis Index
RMSEA	Root Mean Square Error of Approximation
SRMR	Standardized Root Mean Square Residual
VIF	Variance Inflation Factor
