# Supplementary material for: A Theory-Based Approach to Predict Stress Relaxation Behavior Among South Asian Americans: A Cross-Sectional Study
Source: Int J Environ Res Public Health. 2026 Feb 17;23(2):253. doi: 10.3390/ijerph23020253 (PMC12940998; doi:10.3390/ijerph23020253)
Supplement: Supplementary file 1 [file ijerph-23-00253-s001.zip › ijerph-4066470-supplementary.pdf]

MEASURING CHANGE IN RELAXATION BEHAVIOR IRB # ________

Directions: Please mark the response that best describes your position.

Stress is a pervasive issue that affects all of us daily. One of the techniques for dealing with stress is to engage in relaxation behavior for at least 20 minutes daily. Some relaxation techniques are progressive muscle relaxation, visual imagery, autogenic training, yoga, tai chi, meditation, etc. The following questions pertain to the conscious daily pursuit of relaxation behavior. South Asian origin means countries

- ☐ Afghanistan,
- ☐ Bangladesh
- ☐ Bhutan
- ☐ India
- ☐ Maldives
- ☐ Nepal
- ☐ Pakistan
- ☐ Sri Lanka

1. Are you 18 years and older?
   - ☐ Yes
   - ☐ No

If No, then stop taking this survey

…………………………………………………………………………………………………

2. Are you South Asian or of South Asian descent (one of your parents, both parents, one of your grandparents, or both grandparents)?
   - ☐ Yes
   - ☐ No

   If Yes, Please mark all that apply:
   - ☐ Afghanistan
   - ☐ Bangladesh
   - ☐ Bhutan
   - ☐ India
   - ☐ Maldives
   - ☐ Nepal
   - ☐ Pakistan
   - ☐ Sri Lanka

If No, then stop taking this survey

…………………………………………………………………………………………………

3. During the past 24 hours did you engage in conscious pursuit of relaxation behavior other than sleep?
   - ☐ Yes
   - ☐ No

…………………………………………………………………………………………………

4. During the past 24 hours how many minutes of relaxation behavior other than sleep did you practice?

_______ minutes

Items 3-12 are from the Perceived Stress Scale PSS-10

		Never	Almost Never	Sometimes	Fairly Often	Very Often
3.	In the last month, how often have you been upset because of something that happened unexpectedly?	☐	☐	☐	☐	☐
4.	In the last month, how often have you felt that you were unable to control the important things in your life?	☐	☐	☐	☐	☐
5.	In the last month, how often have you felt nervous and "stressed"?	☐	☐	☐	☐	☐
6.	In the last month, how often have you felt confident about your ability to handle your personal problems?	☐	☐	☐	☐	☐
7.	In the last month, how often have you felt that things were going your way?	☐	☐	☐	☐	☐
8.	In the last month, how often have you found that you could not cope with all the things that you had to do?	☐	☐	☐	☐	☐
9.	In the last month, how often have you been able to control irritations in your life?	☐	☐	☐	☐	☐
10.	In the last month, how often have you felt that you were on top of things?	☐	☐	☐	☐	☐
11.	In the last month, how often have you been angered because of things that happened that were outside of your control?	☐	☐	☐	☐	☐
12.	In the last month, how often have you felt difficulties were piling up so high that you could not overcome them?	☐	☐	☐	☐	☐

	Never	Almost Never	Sometimes	Fairly Often	Very Often
**If you start practicing relaxation behavior for 20 minutes daily how often do you feel you may…**					
13. … be healthier.	☐	☐	☐	☐	☐
14. … not feel tired.	☐	☐	☐	☐	☐
15. … be happier.	☐	☐	☐	☐	☐
16. … have more energy.	☐	☐	☐	☐	☐
17. … manage your weight.	☐	☐	☐	☐	☐

	Never	Almost Never	Sometimes	Fairly Often	Very Often
**If you start practicing relaxation behavior for 20 minutes daily how often do you feel you may…**					
18. … have less time for other things.	☐	☐	☐	☐	☐
19. … feel less energetic.	☐	☐	☐	☐	☐
20. … not be able to find time to do it.	☐	☐	☐	☐	☐
21. … still not be able to relax.	☐	☐	☐	☐	☐
22. … feel that others think negatively about you.	☐	☐	☐	☐	☐

	Not At All Sure	Slightly Sure	Moderately Sure	Very Sure	Completely Sure
**How sure are you that you will start practicing relaxation behavior for 20 minutes …**					
23. … daily?	☐	☐	☐	☐	☐
24. … daily while maintaining your other duties?	☐	☐	☐	☐	☐

		Not At All Sure	Slightly Sure	Moderately Sure	Very Sure	Completely Sure
25.	… daily despite any opposition from others?	☐	☐	☐	☐	☐
26.	… daily despite being busy?	☐	☐	☐	☐	☐
27.	… daily despite not enjoying it?	☐	☐	☐	☐	☐

		Not At All Sure	Slightly Sure	Moderately Sure	Very Sure	Completely Sure
**How sure are you that you will be able to…**						
28.	… find a quiet place to practice relaxation?	☐	☐	☐	☐	☐
29.	… eliminate distractions preventing you from relaxing from your physical environment?	☐	☐	☐	☐	☐
30.	… have the necessary resources to practice relaxation?	☐	☐	☐	☐	☐

		Not At All Sure	Slightly Sure	Moderately Sure	Very Sure	Completely Sure
**How sure are you that you can…**						
31.	… direct your emotions/feelings to the goal of practicing relaxation for 20 minutes daily?	☐	☐	☐	☐	☐
32.	… motivate yourself to practice relaxation for 20 minutes daily?	☐	☐	☐	☐	☐
33.	… overcome self-doubt in accomplishing the goal of practicing relaxation for 20 minutes daily?	☐	☐	☐	☐	☐

		Not At All Sure	Slightly Sure	Moderately Sure	Very Sure	Completely Sure
**How sure are you that you can…**						
34.	… keep a self-diary/journal to monitor the goal of practicing relaxation for 20 minutes daily?	☐	☐	☐	☐	☐

35. … be able to practice relaxation for 20 minutes daily if you encounter barriers?	☐	☐	☐	☐	☐
36. … change your plan for practicing relaxation for 20 minutes daily if you face difficulties?	☐	☐	☐	☐	☐

	**Not At All Sure**	**Slightly Sure**	**Moderately Sure**	**Very Sure**	**Completely Sure**
**How sure are you that you can get the help of a…**					
37. …family member to support you with practicing relaxation for 20 minutes daily?	☐	☐	☐	☐	☐
38. …friend to support you with practicing relaxation for 20 minutes daily?	☐	☐	☐	☐	☐
39. …health professional to support you with practicing relaxation for 20 minutes daily?	☐	☐	☐	☐	☐

	**Not At All Likely**	**Somewhat Likely**	**Moderately Likely**	**Very Likely**	**Completely Likely**
**How likely is it that you will…**					
40. … practice relaxation for 20 minutes daily in the upcoming week.	☐	☐	☐	☐	☐
41. … consider practicing relaxation for 20 minutes daily in the upcoming week.	☐	☐	☐	☐	☐
42. … intend to practice relaxation for 20 minutes daily in the upcoming week.	☐	☐	☐	☐	☐

	Not At All Likely	Somewhat Likely	Moderately Likely	Very Likely	Completely Likely

How likely is it that you will…

		Not At All Likely	Somewhat Likely	Moderately Likely	Very Likely	Completely Likely
43.	… practice relaxation for 20 minutes daily from now on.	☐	☐	☐	☐	☐
44.	… consider practicing relaxation for 20 minutes daily from now on.	☐	☐	☐	☐	☐
45.	… intend to practice relaxation for 20 minutes daily from now on.	☐	☐	☐	☐	☐

46. What is your gender?
☐ Male
☐ Female
☐ Other, ________________

47. How old are you today? _______ years

48. What is your nationality?
☐ United States
☐ Other, ________________

49. What is your highest level of education?
☐ Some schooling but not completed high school
☐ Completed high school or GED
☐ Some college
☐ Completed college/ Graduate degree
☐ Post graduate degree
☐ Professional degree

50. What is your marital status?
☐ Married
☐ Divorced
☐ Widowed
☐ Separated
☐ Never married
☐ In a civil union or registered domestic partnership
☐ A member of an unmarried couple

51. Do you work for pay?
☐ No
☐ Yes, How many hours? _________
Which employment sector?
☐ Healthcare
☐ Information Technology

□ Real estate and development
□ Retail
□ Education
□ Government
□ Other ______________

………………………………………………………………………………………………………

52. Do you have health insurance?
□ Yes
□ No

………………………………………………………………………………………………………

53. How long have you lived in the United States? ___________________

………………………………………………………………………………………………………

54. What is your yearly household income?
□ Less than $ 50,000
□ $ 50,001 to $ 100,000
□ $100,001 to $150,000
□ $150,001 to $200,000
□ More than $200,000
□ Prefer not to answer

………………………………………………………………………………………………………

55. Do you smoke cigarettes?
□ No
□ Yes, If so how often
  □ A few times a year
  □ At least once a month
  □ At least once a week
  □ At least once a day

………………………………………………………………………………………………………

56. Do you drink alcohol?
□ No
□ Yes, If so how often
  □ Once in a while
  □ Sometimes
  □ At least once a week
  □ At least once a day

………………………………………………………………………………………………………

57. Have you been diagnosed with any mental illness in the past year?
□ No
□ Yes

………………………………………………………………………………………………………

58. How would rate your work-life balance:
□ Excellent
□ Very Good
□ Good
□ Fair

□ Poor

……………………………………………………………………………………………………

59. How many hours do you sleep (including naps) on a typical day? ___________

60. On a typical day, how many hours do you engage in any form of work (including house chores)? __________

………………………………………………………………………………………………………

61. Which region of the United States do you belong to?

□ Northeast (CT, DC, DE, MA, MD, ME, NH, NJ, NY, PA, RI, VT)

□ Southwest (AZ, NM, OK, TX)

□ West (AK, CA, CO, HI, ID, MT, NV, OR, UT, WA, WY)

□ Southeast (AL, AR, FL, GA, KY, LA, MS, NC, SC, TN, VA, WV)

□ Midwest (IA, IL, IN, KS, MI, MN, MO, ND, NE, OH, SD, WI)

………………………………………………………………………………………………………

Thank you for your time!

SCORING

Total Perceived Stress scores (PSS-10) use a scale: Never (0), Almost Never (1), Sometimes (2), Fairly often (3), Very often (4). Summative scores from items 3-12 are obtained by reversing the scores on the four positive items, e.g., 0=4, 1=3, 2=2, etc., and then summing across all 10 items. Items 6, 7, 9, and 10 are the positively stated items. Possible range: 0-40. A high score indicates more stress. This variable can be used as a covariate.

The construct of advantages: Scale: Never (0), Almost Never (1), Sometimes (2), Fairly often (3), Very often (4). Summative score of Items 13-17. Possible range: 0-20. The high score is associated with the likelihood of initiation of behavior change.

Construct of disadvantages: Scale: Never (0), Almost Never (1), Sometimes (2), Fairly often (3), Very often (4). Summative score of Items 18-22. Possible range: 0-20. A low score is associated with the likelihood of initiation of behavior change.

Subtract the disadvantages score from the advantages score to calculate the **participatory dialogue** construct score. A positive score will be indicative of behavior change.

The construct of behavioral confidence: Scale: Not at all sure (0), slightly sure (1), moderately sure (2), very sure (3), completely sure (4). Summative score of Items 23-27. Possible range 0-20. The high score is associated with the likelihood of initiation of behavior change.

The construct of changes in the physical environment: Scale: Not at all sure (0), slightly sure (1), moderately sure (2), very sure (3), completely sure (4). Summative score of Items 28-30. Possible range 0-12. The high score is associated with the likelihood of initiation of behavior change.

The construct of emotional transformation: Scale: Not at all sure (0), slightly sure (1), moderately sure (2), very sure (3), completely sure (4). Summative score of Items 31-33. Possible range 0-12. The high score is associated with the likelihood of sustenance of behavior change.

The construct of practice for change: Scale: Not at all sure (0), slightly sure (1), moderately sure (2), very sure (3), completely sure (4). Summative score of Items 34-36. Possible range 0-12. The high score is associated with the likelihood of sustenance of behavior change.

The construct of changes in the social environment: Scale: Not at all sure (0), slightly sure (1), moderately sure (2), very sure (3), completely sure (4). Summative score of Items 37-39. Possible range 0-12. The high score is associated with the likelihood of sustenance of behavior change.

For **modeling initiation, the** dependent variable can be a summative score of Items 40-42: not at all likely (0), somewhat likely (1), moderately likely (2), very likely (3), and completely likely (4), with a range of 0-12 and multiple regression can be used. For **modeling sustenance, the** dependent variable can be Item 43-45: not at all likely (0), somewhat likely (1), moderately likely (2), very likely (3), and completely likely (4), with a range of 0-12 and multiple regression can be used.

Flesch Reading Ease: 63.7
Flesch-Kincaid Grade Level: 5.9

©Manoj Sharma

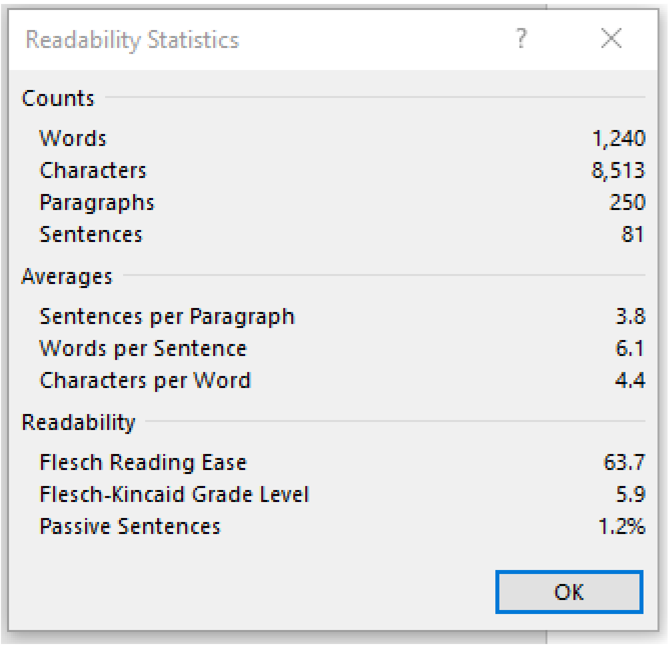
Readability Statistics
?
Counts
Words 1,240
Characters 8,513
Paragraphs 250
Sentences 81
Averages
Sentences per Paragraph 3.8
Words per Sentence 6.1
Characters per Word 4.4
Readability
Flesch Reading Ease 63.7
Flesch-Kincaid Grade Level 5.9
Passive Sentences 1.2%
OK
